# Unraveling the Potential of γ-Aminobutyric Acid: Insights into Its Biosynthesis and Biotechnological Applications

**DOI:** 10.3390/nu16162760

**Published:** 2024-08-19

**Authors:** Lei Zhu, Zhefeng Wang, Le Gao, Xiaoyi Chen

**Affiliations:** 1 School of Biological Engineering, Dalian Polytechnic University, Dalian 116034, China; zhulei@tib.cas.cn; 2 Tianjin Institute of Industrial Biotechnology, Chinese Academy of Sciences, National Technology Innovation Center for Synthetic Biology, Tianjin 300308, China; wangzhefeng22@mails.ucas.ac.cn

**Keywords:** catalytic mechanism, crystal structure, GABA, GAD, microorganism, physiological effects

## Abstract

γ-Aminobutyric acid (GABA) is a widely distributed non-protein amino acid that serves as a crucial inhibitory neurotransmitter in the brain, regulating various physiological functions. As a result of its potential benefits, GABA has gained substantial interest in the functional food and pharmaceutical industries. The enzyme responsible for GABA production is glutamic acid decarboxylase (GAD), which catalyzes the irreversible decarboxylation of glutamate. Understanding the crystal structure and catalytic mechanism of GAD is pivotal in advancing our knowledge of GABA production. This article provides an overview of GAD’s sources, structure, and catalytic mechanism, and explores strategies for enhancing GABA production through fermentation optimization, metabolic engineering, and genetic engineering. Furthermore, the effects of GABA on the physiological functions of animal organisms are also discussed. To meet the increasing demand for GABA, various strategies have been investigated to enhance its production, including optimizing fermentation conditions to facilitate GAD activity. Additionally, metabolic engineering techniques have been employed to increase the availability of glutamate as a precursor for GABA biosynthesis. By fine-tuning fermentation conditions and utilizing metabolic and genetic engineering techniques, it is possible to achieve higher yields of GABA, thus opening up new avenues for its application in functional foods and pharmaceuticals. Continuous research in this field holds immense promise for harnessing the potential of GABA in addressing various health-related challenges.

## 1. Introduction

In 1950, Roberts and Frankel made a significant discovery using two-dimensional paper chromatography, where they identified a large number of previously unrecognized ninhydrin reaction points in the brains of mammals, including humans, mice, and rabbits. These compounds were later conclusively identified as γ-aminobutyric acid (GABA) [1]. It is worth noting that most microorganisms producing GABA share a common characteristic—they thrive under acidic conditions, which may be attributed to their acid-resistance capabilities [2]. Further research has revealed that GABA can stimulate microbial spore germination and promote growth [3]. In the plant kingdom, GABA plays a crucial role in maintaining the carbon and nitrogen pools within plant cells, as well as participating in nitrogen metabolism. Additionally, GABA can promote seed germination, accelerate maturation, and participate in maintaining redox balance within plant cells, as well as alleviating abiotic stress [4,5]. Therefore, GABA can be applied in agriculture as a novel plant growth regulator to improve agricultural productivity and product quality. In animals, GABA also holds significant importance, with various functions such as reducing blood pressure [6], promoting dopamine release [7], regulating heart rate and protecting the heart [8], balancing blood sugar levels [9,10,11], protecting the liver [12], inhibiting the proliferation and metastasis of cancer cells [13], and exhibiting numerous other physiological effects including anti-inflammatory properties, antioxidant properties, and promoting growth and development [14,15]. Due to its significant role in modulating neuronal excitation, researchers are actively engaged in developing psychotropic targeting drugs with GABA [16,17,18]. Consequently, GABA has promising prospects in the pharmaceutical and functional food fields.

Currently, there are two main methods for GABA production: the chemical method and the biological conversion method. When compared to chemical methods, biosynthetic methods offer several advantages. For instance, they involve mild reaction conditions, a simple process, and lower costs. However, the key component of the biological conversion method is glutamic acid decarboxylase (GAD). GAD is an amino acid decarboxylase that relies on pyridoxal 5′-phosphate (PLP) and can exist as either an inactive enzyme protein or an active holoenzyme [19]. GAD specifically catalyzes the conversion of L-glutamic acid or L-glutamate salt into GABA [20]. GAD is widely present in various organisms, ranging from single-celled organisms to mammals. Among these organisms, microorganisms offer advantages such as simple growth conditions, short growth cycles, and unique metabolic processes, making them the primary source of biological enzymes. Moreover, utilizing microorganisms for enzyme production is not easily constrained by resources, environment, or space, and genetic modification can be readily performed [21]. However, variations in the structure and function of GADs exist among different sources. Therefore, the directional modification of existing GADs using protein engineering, bioinformatics, genetic engineering, and other technologies plays a crucial role in the production of cost-effective GADs with enhanced properties.

Protein engineering, built upon the foundations of genetic engineering and molecular biology, integrates various disciplines such as protein crystallography, computer-aided design, and artificial intelligence. While enzymes hold tremendous potential in the biocatalytic industry, naturally occurring enzymes often possess inherent limitations such as low activity, poor stability, and a limited substrate range. Protein engineering allows for the predictable modification of protein structures to enhance specific functional properties [22]. By thoroughly understanding and mastering the principles governing protein structure and function, it becomes possible to create proteins with novel characteristics. For instance, Wahab et al. employed protein engineering to replace exposed amino acid residues opposite the active center of xylanase with lysine, resulting in a stable cross-linked mutant xylanase when bovine serum protein was added. This mutant xylanase exhibited 1.09 times higher stability compared to the cross-linked recombinant xylanase with bovine serum protein. Additionally, the mutant xylanase could be reused for up to eight cycles, demonstrating excellent repeatability [23]. However, GADs derived from different sources exhibit distinct structures and functions, and there are limited reports on their relationships. Therefore, this article primarily reviews the structures of GADs from various sources and aims to comprehend their catalytic mechanisms. This article also explores effective strategies for utilizing microbial fermentation to produce GABA from aspects such as optimization of fermentation conditions, metabolic engineering, and genetic engineering. Furthermore, various physiological functions of GABA were also discussed.

## 2. Source of GAD

GAD is widely distributed in microorganisms, animals, and plants, and its role in the central nervous system of animals has been a topic of extensive research, as its substrates and products are neurotransmitters with opposing functions [24]. The enzymatic properties of GAD differ significantly among various sources. Generally, microbial GAD exhibits optimal activity under acidic conditions, with a pH range of 4.0 to 5.0 (Table 1). Plant-derived GAD exhibits optimal activity under mildly acidic conditions, generally slightly higher than microbial GAD, with an optimal pH of approximately 5.5 to 6.0 (Table 2). GAD from animal sources typically shows optimal activity at neutral pH (Table 3). Additionally, the optimal reaction temperature for GAD varies depending on its source. The optimal reaction temperature for microbial GAD can vary considerably. Terrestrial plant-derived GAD displays peak activity around 40 °C, while terrestrial animal GAD typically functions best at 37 °C. In contrast, aquatic organisms, both plant and animal, generally exhibit optimal GAD activity at 30 °C or lower. These observations suggest that the enzymatic properties of GAD from different sources are closely related to the growth conditions of its host organisms.

## 3. The Catalytic Mechanism of GAD

**Figure 1:**
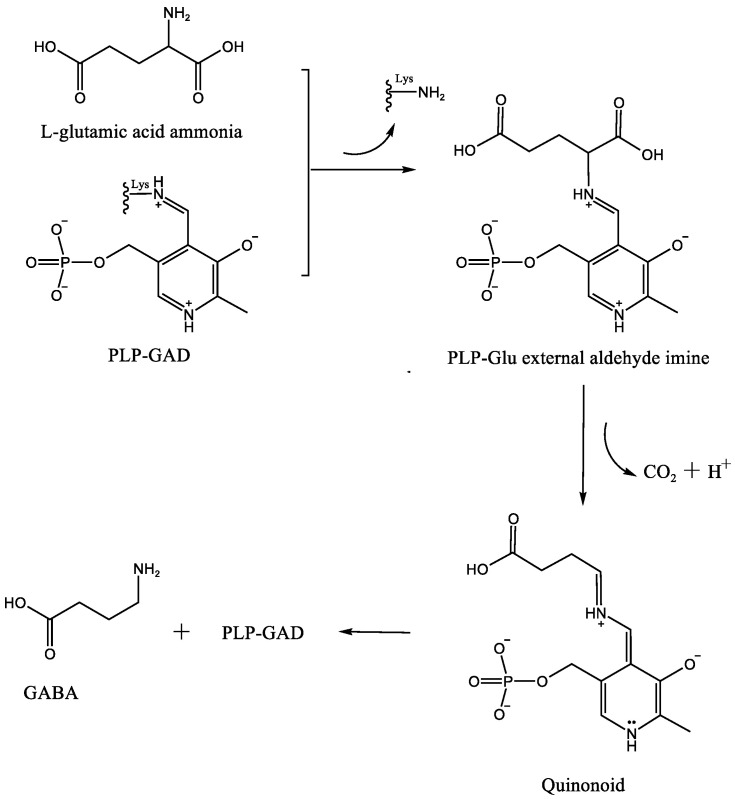
The catalytic process of GAD [54].

GAD is an enzyme that relies on pyridoxal phosphate (PLP) as an essential cofactor. PLP serves as a cofactor for numerous enzymes involved in transamination, decarboxylation, racemization, aldol cleavage, and other reactions [51,52]. In the absence of a substrate, the 4-formyl group of PLP interacts with a conserved lysine residue in the active site of GAD, forming a covalent bond to create an internal methylene structure known as an aldimine or Schiff base. When the substrate L-glutamic acid enters the active site of GAD, PLP forms an external aldehyde imine structure with the substrate. The conjugated system in this structure extends from the pyridine nitrogen to the α-carbon of glutamic acid. The nitrogen atoms in the pyridine ring and Schiff base exhibit strong electrophilic properties, which are further enhanced in their protonated state. As a result, the electron density around the α-carbon decreases, leading to a decrease in bond energy on the α-carbon. This, in turn, facilitates the catalytic decarboxylation reaction by GAD, forming a quinone intermediate. The conjugated system formed by the Schiff base and pyridine ring helps stabilize these quinone intermediates. Subsequently, protonation and transaldimination of the lysine residue in the active site lead to the regeneration of the aldimine within PLP-GAD and the release of the product, γ-aminobutyric acid (GABA) (Figure 1) [53,54].

## 4. The Structural Characteristics of GAD from Different Sources

### 4.1. Microbial GAD

#### 4.1.1. GAD of *E. coli*

Currently, GAD has been identified in various bacteria and fungi, with *E. coli* being the most extensively studied. In *E. coli*, there are two genes, gadA and gadB, encoding this enzyme. The protein products of these genes differ by only five amino acid residues, four of which are located in the N-terminal region. Together with the Glu/GABA reverse transporter encoded by gadC, these enzymes form the acid-tolerant system of *E. coli*, enabling the bacteria to survive in highly acidic environments (pH < 2) for at least two hours. Capitani et al. demonstrated that at a neutral pH, the enzymes exist in the cytoplasm, but as the pH decreases, they adsorb onto the cell membrane [55,56].

**Figure 2:**
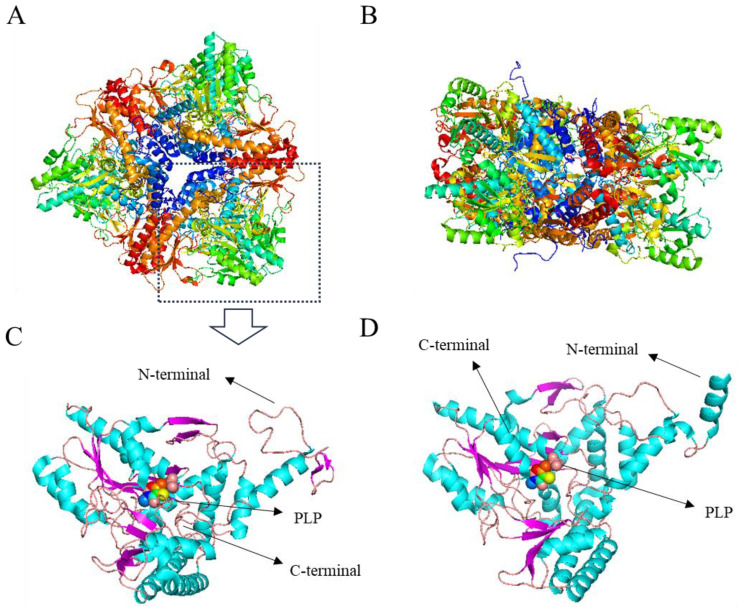
Crystal structure of *E. coli* GAD. (**A**) *E. coli* GAD hexamer surface at neutral pH; (**B**) *E. coli* GAD hexamer side at neutral pH; (**C**) single subunit structure at neutral pH; (**D**) single subunit structure at acidic pH [56].

The 330 kDa *E. coli* GAD is a hexameric protein consisting of three dimers. The hexamers are arranged in two layers, each containing three subunits, with each dimer contributing one subunit to each layer (Figure 2A,B). At a neutral pH, the residues 3–15 of all subunits do not exhibit any regular secondary structure (Figure 2C), and each subunit adopts a distinct conformation. As the pH decreases, there are no significant changes in the overall structure of the hexamer, but each subunit undergoes significant conformational changes in the residues 3–15, transitioning from an irregular structure to an α-helix structure (Figure 2D).

**Figure 3:**
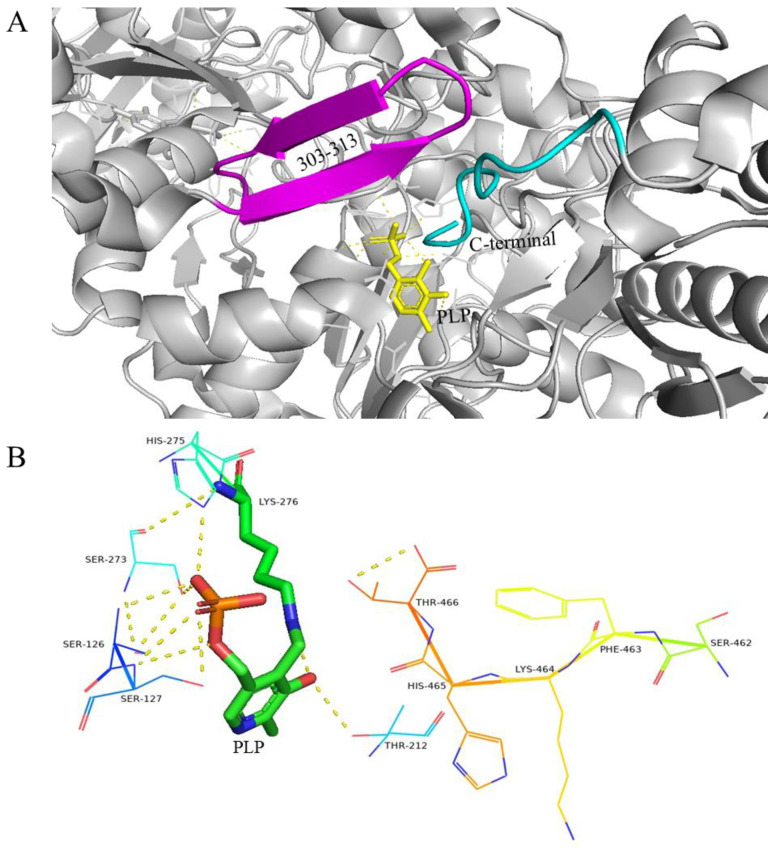
(**A**) Conformation of *E. coli* GAD active centers at neutral pH; (**B**) PLP and nearby residues, yellow dashed lines represent hydrogen bonds, PLP and lysine residues are rod-shaped, and other nearby residues are linear [57].

At a neutral pH, residues 452–466 at the C-terminal region adopt a well-defined crystalline structure that extends towards the interior of the active funnel, reaching the substrate binding site. Simultaneously, the β-hairpin conformation formed by residues 300–313 interacts with the C-terminal residue of another subunit in the dimer, creating steric hindrance and preventing the active center from binding to the substrate (Figure 3A) [57]. The interaction between PLP and neighboring amino acid residues plays a crucial role in supporting the catalytic activity of PLP cofactors. These amino acid residues assist in stabilizing the phosphate and pyridine rings of PLP cofactors, confining them to the active sites (Figure 3B) [58].

#### 4.1.2. GAD of Lactobacillus Brevis CGMCC 1306

In contrast to *E. coli*, both the inactive form (pH 7.0) and active form (pH 4.8) of *Lactobacillus brevis* GAD (LBGAD) exist as dimers, consisting of 468 residues with a molecular weight of approximately 108kDa. The cofactor PLP forms a Schiff base with Lys279, while residues Ser126, Ser127, Cys168, Ile211, Ser276, His278, and Ser321 stabilize PLP in the active center. The crystal structure of the LBGAD monomer resembles that of *E. coli* GAD, featuring an N-terminal helix. The PLP binding domain comprises a seven-chain mixed β-sheet that forms a parallel chain, with residues 287–293 forming an antiparallel chain surrounded by seven α-helices. The flexible ring (residues 308–312), located near the substrate binding site, actively participates in the catalytic reaction, with the conserved residue Tyr308 playing a crucial role in the decarboxylation of L-glutamic acid [59].

#### 4.1.3. GAD of *Bacteroides thetaiotaomicron*

**Figure 4:**
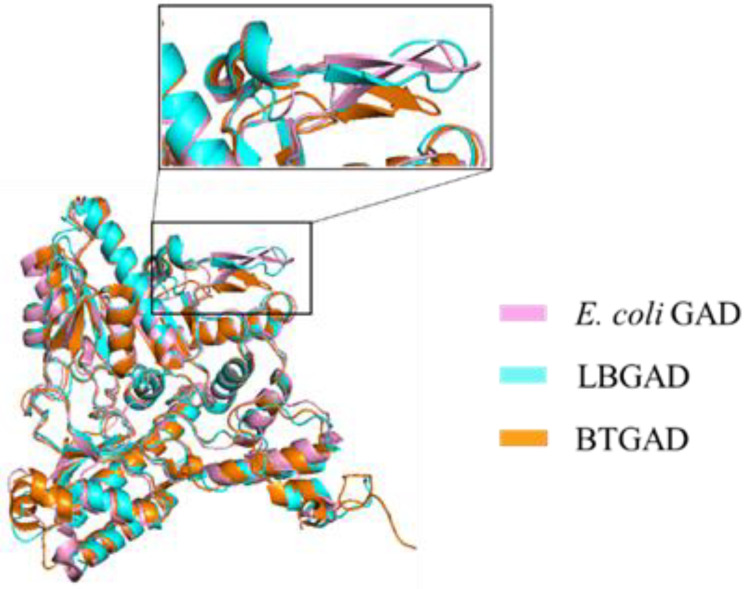
Structural comparison between BTGAD and different GADs.

The gadB gene of *Bacteroides thetaiotaomicron* (BTGAD) encodes a protein consisting of 481 amino acids with a molecular weight of approximately 54.854 kDa. BTGAD exhibits low homology with GAD sequences from other sources, with a consistency of 42.5% to the GAD sequence of *E. coli* and 39.6% to the LBGAD sequence. However, their monomer structures are similar. The BTGAD monomer is also composed of three structural domains: N-terminal (residue 1–51), PLP binding domain (residue 52–359), and C-terminal (residue 360–481). The pH-dependent conformational changes of N-terminal residues 1–24 and C-terminal residues 465–481 in BTGAD are consistent with *E. coli* GAD [57]. The cofactor PLP forms a Schiff base with Lys274 in BTGAD and forms hydrogen bonds and hydrophobic interactions with surrounding residues to stabilize the active center. The corresponding β-hairpin in BTGAD is positioned closer to the substrate binding site compared to other GADs (Figure 4), resulting in more pronounced conformational changes. Additionally, BTGAD exhibits significant pH-dependent oligomeric changes. Studies have demonstrated that GAD exhibits different oligomeric states and absorbance spectra at different pH levels. At pH 4.4, BTGAD exists as hexamers, while at pH 5.8 and 7.0, it exists as a mixture of hexamers and dimers. As the pH increases, the proportion of hexamers decreases, while the proportion of dimers increases. At pH 8.0, BTGAD exists solely as dimers. Hexamers represent the active form of BTGAD, with BTGAD activity nearly absent above pH 5.6 [32].

### 4.2. Plant GAD

GAD has been identified in a wide range of plant species, and the structure of GAD from Arabidopsis thaliana has been elucidated. The structure of Arabidopsis thaliana glutamic acid decarboxylase (AtGAD1) is similar to that of bacterial GADB, forming a hexameric structure through the aggregation of three dimers. Additionally, they share 39% sequence identity [60]. The N-terminal domain (residues 1–57) of AtGAD1 plays an important role in the formation and stability of dimers and hexamers. There is a significant difference in the functional modules of AtGAD1 and GADB at the N-terminal. The residues 1–15 of AtGAD1 are disordered, while at low pH, residues 3–15 of GADB form an α-helix structure. The large structural domain (residues 58–347) contains cofactor binding sites. The small structural domain has four β-folded sheets and three α- helix. Its C-terminal residue connects to CaMBD and affects enzyme activity in a Ca^2+^/CaM-dependent manner [61,62].

The binding of active sites and cofactors in AtGAD1 exhibits strong conservation. In AtGAD1, PLP forms a covalent linkage with Lys277 through a Schiff base. The pyridine ring is positioned between Gln163 and Ala246, while the nitrogen of Asp244 and the pyridine ring form a salt bridge. Other residues involved in cofactor binding (Phe63, Ser127, Thr213, His276) are also conserved in AtGAD1 and occupy the same structural positions as in GADB. Notably, the position in AtGAD1 corresponding to Thr62 in GADB is substituted with serine Ser62, which can serve as a hydrogen donor when the substrate binds to the active center [62].

Plant-derived GAD possesses a unique characteristic compared to other sources of GAD, which is the presence of a calmodulin (CaM) binding site (CaMBD) at its C-terminus. This CaMBD enables plant GAD to sense calcium ion signals, providing an additional regulatory mechanism for responding to cytoplasmic calcium. Thus, in plants, GAD activity can be regulated by two mechanisms: acidic pH and Ca^2+^/CaM [63,64]. Calcium regulatory proteins are activated by a multifunctional Ca^2+^ signal sensor that responds to various extracellular stimuli (such as hormones and neurotransmitters), leading to an increase in intracellular Ca^2+^ concentration [65]. At low Ca^2+^ concentrations, the catalytic activity of CaM is inhibited by its self-inhibitory region interaction. However, upon stimulation by extracellular signals, the intracellular Ca^2+^ concentration rises, causing CaM to bind to Ca^2+^, undergo conformational changes, and subsequently interact with the CaMBD of the target protein, thereby affecting numerous physiological processes [66,67].

### 4.3. Animal GAD

In animals, glutamate (Glu) serves as the primary excitatory neurotransmitter, playing a crucial role in various behaviors, including learning, memory, and aggression [68,69]. However, excessive glutamate levels can have toxic and neurodegenerative effects on neurons. Conversely, γ-aminobutyric acid (GABA) functions as the key inhibitory neurotransmitter and is synthesized from glutamate through the catalytic activity of GAD [70,71]. As a result, GAD plays a vital role in maintaining the balance between Glu and GABA concentrations [72]. The mechanism of action of GAD in animals has garnered significant attention as a prominent research topic.

**Figure 5:**
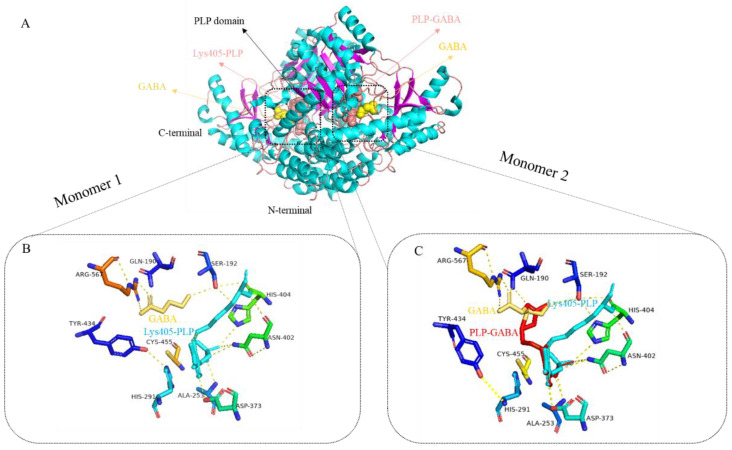
(**A**) Crystal structure of dimer GAD67; (**B**,**C**) The PLP and residues near the active site in the two monomeric subunits of GAD67 [73].

There are two primary subtypes of GAD in animals, known as GAD67 and GAD65. GAD67 forms a specialized dimer structure, comprising three distinct domains: the N-terminal domain, the PLP domain, and the C-terminal domain. The N-terminal domain consists of two parallel α-helical spirals. The PLP domain is composed of nine α-helices surrounding a β-folded sheet consisting of nine parallel strands. The C-terminal domain contains three α-helices and four reverse parallel β-folds (Figure 5A). At each active site, Schiff bases are formed between PLP and lysine residues (Lys405). In monomer 1, the active site contains a GABA molecule (Figure 5B). In monomer 2, two disordered structural conformations of GABA were observed at the active center. In one conformation, GABA forms a salt bridge with Arg567 and hydrogen bonds with Gln190. In the other conformation, the continuous electron density between PLP and GABA suggests the presence of PLP-GABA intermediates (Figure 5C) [73].

It is worth noting that each active site is essentially covered by a loop ring (residues 432–442, referred to as the “catalytic ring”) that extends from another monomer. This catalytic ring causes the conserved residue Tyr434 to come into close proximity with His291 and GABA through base stacking [74]. In monomer 1, the hydroxyl group of Tyr434′s side chain forms a hydrogen bond with the Nε2 residue of His291 (Figure 5B). In monomer 2, Tyr434 undergoes a conformational change, and the hydrogen bond of the side chain’s hydroxyl group is now bonded to the main chain N of His291 (Figure 5C).

GAD65 and GAD67 share a structural similarity of 71%, and GAD65 adopts the same folding and dimer arrangement as GAD67. In GAD65, PLP binds covalently to residue Lys396 at the two active sites. Each active center of GABA in GAD65 exhibits two disordered conformations, but unlike GAD67, there is no electron density indicating the presence of PLP-GABA intermediates. Additionally, the corresponding catalytic ring region in GAD65 is disordered, resulting in fully exposed active sites [73].

Interestingly, while most neurotransmitters are synthesized by a single enzyme, GABA is catalyzed by two isomers, GAD67 and GAD65. GAD67 is constantly active, maintaining the metabolic balance of Glu/GABA in the body, while GAD65 is mostly inactive and only becomes activated when the body requires GABA urgently. Furthermore, in animal models, the expression levels of GAD are closely correlated with those of parvalbumin. Overexpression of parvalbumin leads to upregulation of GAD65/GAD67 expression, while silencing parvalbumin results in the opposite effect [75]. GAD levels are regulated by neural activity and undergo changes in various psychiatric disorders [76].

## 5. Multiple Strategies for High Production of GABA in Microbial Cell Factories

GABA, a non-protein amino acid with four carbon atoms, plays a critical role in various physiological and biochemical metabolic processes in animals, plants, and microorganisms. It serves as the primary inhibitory neurotransmitter in the animal nervous system and has extensive applications in pharmaceuticals and functional foods [77]. Initially, GABA was synthesized using chemical methods; however, these methods were not widely accepted due to their significant environmental impact [78]. Different treatments have been explored to elevate GABA levels in plants. For example, salt treatment [79] of wheat and soybeans, anaerobic treatment [80] of tea leaves, and low-temperature [81] treatment of fruits and vegetables can all activate GAD, consequently leading to increased GABA accumulation in plants. Nevertheless, plant-based GABA enrichment is predominantly used in food processing, but its limited yield hinders its application in other areas.

Microbial synthesis technology for GABA is considered a more promising and environmentally friendly approach. To meet the growing market demand, it is imperative to develop cost-effective methods for GABA production.

### 5.1. GABA Pathway

**Figure 6:**
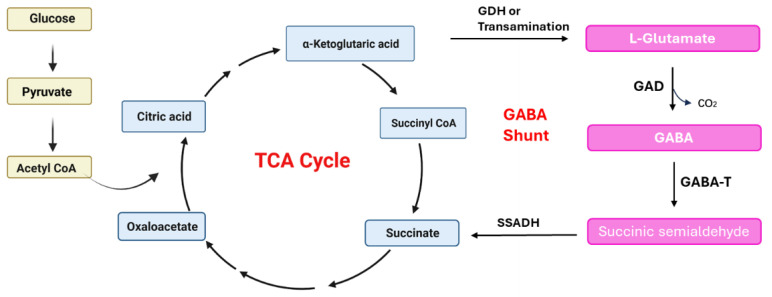
Main metabolic pathways of GABA [82].

Due to the presence of GAD and PLP cofactors, GABA can be synthesized through irreversible decarboxylation reactions in various microorganisms, including bacteria, yeast, and fungi. The mechanism of GABA production in bacteria is illustrated in Figure 6. Microorganisms have the ability to produce GABA by directly utilizing L-glutamic acid as a substrate. Alternatively, glucose can be converted to pyruvate through glycolysis, which is then transformed to acetyl-CoA and enters the TCA cycle to produce α-Ketoglutaric acid. α-Ketoglutarate is catalyzed by glutamate dehydrogenase (GDH) to form L-glutamic acid, which is subsequently decarboxylated by GAD to generate GABA. Additionally, microorganisms possess a pathway to produce GABA by bypassing certain key steps in the TCA cycle. GABA within the mitochondrial matrix undergoes the action of GABA transaminase (GABA-T) to produce succinate semialdehyde (SSA), which is further oxidized by succinic acid semialdehyde dehydrogenase (SSADH) to succinic acid, ultimately resulting in the production of α-Ketoglutaric acid [82]. While SSADH is not directly involved in GABA biosynthesis, it catalyzes the first step in the degradation of GABA, and its enzymatic activity is essential for preserving intracellular GABA balance. Succinate serves as an electron donor in the mitochondrial electron transfer chain and plays a crucial role in the TCA cycle. GAD is present in the cytoplasm, while GABA-T and SSADH are located in the mitochondria [78]. The primary function of the GABA shunt is to generate GABA. However, the precursor molecules α-Ketoglutaric acid and succinic acid, which are intermediate products of the TCA cycle, also play a vital role in cell growth. This creates a competition between cell growth and the production of target chemicals, such as GABA. If the metabolic process is excessively biased towards GABA production, it can result in a decrease in bacterial cells, leading to inefficient fermentation. To achieve efficient fermentation and optimal GABA production, it is necessary to establish a suitable metabolic regulatory network that addresses the trade-off between cell growth and GABA production.

Soma et al. developed a novel metabolic regulatory network in *E. coli* to achieve metabolic state-switching during glucose fermentation for GABA production. Prior to IPTG induction, the bacterial cells were in a growth mode, focusing on accumulating biomass. After induction, the overall metabolic pathway was redirected towards GABA production, leading to a three-fold increase in total GABA production titer and yield [83]. Liang et al. constructed a growth phase-dependent autonomous bifunctional genetic switch (GABS) in *C. glutamicum* to reengineer the metabolic pathway for GABA synthesis. This not only reduced competition among biosynthetic modules but also improved the conversion rate from glycerol to GABA, reaching a yield of 0.4 g/g glycerol [84].

### 5.2. Directed Evolution of GAD

The structure and activity of enzymes are intimately linked. Minor alterations in enzyme structure can have a significant impact on its catalytic activity and stability. Enzyme molecular directed evolution is a relatively new protein-engineering strategy that has emerged in recent years. It allows for the simulation of in vitro evolution processes (such as random mutation, recombination, and directed screening of coding genes). This approach enables the generation of enzymes with improved or novel functions, known as evolutionary enzymes. In just a few days or weeks, it can replicate the natural evolutionary process that would normally take thousands of years. A thorough understanding of the GAD enzyme structure allows us to engineer the enzyme through directed evolution and targeted modifications, enhancing its catalytic activity and stability.

Most microorganisms’ GADs exhibit optimal catalytic activity under acidic conditions (pH 4.0–5.0), with activity sharply decreasing as the pH approaches neutral levels. Lyu et al. developed a mutant ${GADB}_{∆C11}$ by removing 11 residues from the C-terminus of the GAD in *Lactobacillus brevis*. This mutant maintained high catalytic activity at pH 6. Additionally, they performed site-directed mutagenesis on four amino acids in GADB_∆C11, resulting in a significantly improved catalytic activity in the mutant ${GADBC}_{∆C11}^{K17T/D294G/E312S/Q346H}$. When the mutated gene was expressed in *E. coli* without pH control, the GABA production concentration reached 270.42 g/L after 7.5 h of fermentation [85]. Yang et al. also engineered a mutant GAD enzyme (E313S) from Lactobacillus plutarum LC84, which exhibited a 62.4% increase in activity compared to the wild-type enzyme at the optimal pH. Moreover, its enzyme activity was five times higher than that of the wild-type enzyme at pH 6.2 [86]. For industrial applications and large-scale production, it is often necessary for enzymes to possess stronger heat resistance. However, the thermal stability of most microbial GADs is poor. Fan et al. conducted saturation site-directed mutagenesis on the N-terminus of *E. coli* GADB to address this issue. The triple mutant (M6, Gln5Ile/Val6Asp/Thr7Gln) demonstrated enhanced thermal stability, with its half-life at 45 °C increasing by 5.6 times [87].

### 5.3. Optimization of Fermentation Parameters

During the fermentation process, the production of GABA is influenced by several factors, including pH, temperature, carbon and nitrogen sources, initial concentration of L-glutamate, PLP addition, and the presence of trace elements.

#### 5.3.1. Carbon and Nitrogen Sources

The composition of the culture medium plays a crucial role in GABA production, particularly the carbon and nitrogen sources. Different microorganisms exhibit varying preferences for carbon sources. For instance, certain strains of Levilactobacillus show a preference for sucrose over glucose as a substrate. In a study by Li et al., the effects of different carbon sources (glucose, sucrose, fructose, sodium acetate, soluble starch) and nitrogen sources (yeast powder, peptone, beef extract, urea, potassium nitrate) on GABA production in *Levilactobacillus* sp. LB-2 were investigated. The highest GABA content was observed when sucrose was added at a concentration of 35 g/L and peptone at 32 g/L [88]. Additionally, Zhang et al. discovered that the addition of peptone significantly increased GABA production in *K. marxianus* C21, whereas glucose and vitamin B6 had no significant impact on its yield [27].

#### 5.3.2. pH

The optimal pH for glutamate decarboxylase (GAD) varies among different sources, but it is generally observed to be under acidic conditions. This pH value is typically below the neutral pH for cell cultivation. Park et al. enabled *E. coli* to withstand acidic conditions by introducing DR1558 (a *Deinococcus radiodurans* response regulator) and subsequently conducted high-density fermentation at pH 5 through HCl adjustment, which yielded a 1.7-fold increase in GABA production compared to a strain lacking DR1558 [89].

#### 5.3.3. Initial Concentration of L-Glutamate

Glutamate is the preferred substrate for GABA production, as it can be directly decarboxylated by GAD to form GABA. Interestingly, under low concentrations of glutamate in the culture medium, the yield of GABA can be increased. Conversely, high concentrations of glutamate can lead to increased osmotic pressure in the cells, disrupting bacterial metabolism and inhibiting both bacterial growth and GAD activity [90]. In a study by L et al., the effects of different concentrations of monosodium glutamate on the growth and GABA production of *B. subtilis* BBEL02 cells were investigated. It was found that when 50 g/L of monosodium glutamate was added, the GABA concentration reached its maximum [91].

#### 5.3.4. Temperature

The cultivation temperature plays a crucial role in GABA production. It impacts the catalytic activity of organisms and influences the thermodynamic equilibrium of the reaction. The ideal temperature range for GABA production is typically between 30 and 37 °C [82]. In a study by Villegas et al., the yield of GABA produced by *Lactobacillus brevis* CRL 1942 was investigated at various temperatures ranging from 22 °C to 37 °C. The optimal condition for GABA production was found to be at 30 °C. However, when the temperature exceeded 45 °C, the strain exhibited limited growth and GABA accumulation was not observed [92].

#### 5.3.5. Addition of Pyridoxal-5′-Phosphate (PLP) and Trace Elements

GAD is an enzyme that relies on pyridoxal-5′-phosphate (PLP) as a cofactor, and the addition of PLP during fermentation can influence GABA production. Studies have shown that both a deficiency and an excess of PLP can disrupt amino acid metabolism and hinder cell growth [83]. Moreover, the inclusion of trace elements can act as activators for GAD enzymes, thereby impacting the yield of GABA. Research has indicated that the production of GABA is unaffected by NH_4_^+^, K^+^, and Na^+^. However, the presence of Co^2+^, Ca^2+^, and Mg^2+^ can enhance GAD activity. On the other hand, Cu^2+^, Fe^2+^, Fe^3+^, and Ag^+^ act as inhibitors, hindering GABA production. Larute et al. discovered that Cl- can significantly improve the ability of *Lactococcus lactis* NCDO 2118 to produce GABA, leading to a 4~10-times increase in GABA concentration [32,93].

In summary, various strategies such as genetic engineering and metabolic engineering can enhance GAD enzyme activity and GABA production to different extents. Researchers often combine multiple approaches to increase GABA production. For instance, Yang et al. generated a mutant GADBM4-2 using error-prone PCR, which exhibited excellent performance at both acidic and neutral pH levels. By introducing the central regulatory factor GADE of the acid-resistant system and an enzyme that does not rely on DXP for PLP biosynthesis into recombinant *E. coli*, they established a self-supply system for PLP [94]. This resulted in a final GABA titer of 307.5 ± 5.94 g/L in a 5L bioreactor without the addition of exogenous cofactors [95].

At present, most studies on GABA production are confined to the laboratory scale. Compared to small-scale laboratory fermentation, large-scale industrial production of GABA involves more engineering considerations, such as fermenter design, aeration, and agitation. Consequently, high-yielding strains identified in laboratory settings often exhibit decreased yield and instability when scaled up for industrial production [96]. Therefore, despite advancements in laboratory research, translating GABA production from the laboratory to industrial scale remains challenging.

## 6. GABA Regulates Physiological Metabolism

**Figure 7:**
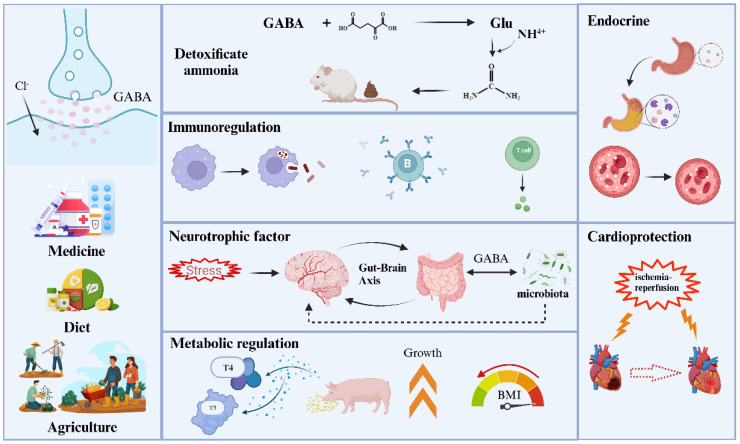
The effects of GABA on animal bodies.

GABA, a naturally occurring non-protein functional amino acid, serves as a vital inhibitory neurotransmitter within the central nervous system of mammals, exerting regulatory influence over various bodily functions [97]. As research progresses, the physiological role of GABA has become increasingly elucidated, evolving into a novel functional factor extensively employed in medicine, food healthcare, and agricultural industries (Figure 7).

### 6.1. Animal Feeding

For instance, in neonatal broiler chickens, GABA stimulates the secretion of gastric acid and digestive enzymes while inhibiting the release of cholecystokinin (CCK) and attenuating the negative feedback effect of digestive chyme on feeding [98]. Consequently, it enhances animal feed intake. Shuye et al. documented that administering GABA within a specific dosage range into various brain regions of animals substantially boosts animal feeding with a dose-dependent impact on rats [99], and this is consistent with the results of experiments on sheep, where the dietary addition of GABA significantly increased feed intake of sheep (*p* < 0.05) [100]. In addition, a certain dose of GABA can also improve the feed intake and growth performance of pigs, but the optimal addition amount of different tests is not consistent, and the appropriate addition amount should be considered for different growth stages of pigs in practical application [100,101,102].

### 6.2. Endocrine and Gastrointestinal Function

As a neurotransmitter or modulator, GABA exerts a broad spectrum of effects in the peripheral autonomic nervous system of vertebrates. GABA, along with its synthesizing and degrading enzymes, is also present in the thyroid and pancreatic islets, with the thyroid capable of accumulating exogenous GABA [103]. Xu et al. observed a significant concentration-dependent promotion of gastric acid secretion by GABA in whole stomach specimens cultured in vitro from mice [104].

GABA is extensively distributed throughout the gastrointestinal (GI) tract, with Nakajima reporting its regulatory effects on gastrointestinal smooth muscle cells, endocrine cells, and non-endocrine cells in rats [105]. Currently, a substantial body of evidence supports the potential role of GABA in GI function regulation [106]. Zhang et al.’s study demonstrated GABA’s efficacy in controlling the weight of mice with type 2 diabetes, maintaining blood glucose balance, improving glucose and lipid metabolism, and influencing their intestinal flora [107]. Zhao et al. reported that GABA supplementation promotes intestinal function and affects intestinal microbiota in piglets [108].

### 6.3. Dietary Supplement

The addition of an appropriate amount of GABA to animal diets can significantly increase the secretion of triiodothyronine (T3) and tetraiodothyronine (T4), indicating GABA’s involvement in regulating body metabolism [109]. In the modern livestock and poultry industry, livestock and poultry grow fast, with vigorous metabolisms. Due to the lack of sweat glands in their skin, hot and high temperatures in the growth of livestock lead to more obvious poultry damage, causing huge economic losses to livestock and poultry breeding [110]. Al Wakeel’s research demonstrated that GABA can alleviate heat stress in commercial broiler chickens through hormone and metabolic regulation, such as affecting the levels of T3, glucose transporter type 2 (GLUT2), and heat shock protein 70 (HSP70) [111].

As an inhibitory neurotransmitter of the central nervous system, GABA can inhibit the integration of the respiratory center, reduce the respiratory rate, and maintain the regulatory role of the central system on the body. It has been proved that adding GABA to the drinking water of broilers can reduce the caloric production, metabolic rate and relieve heat stress of chickens [112].

And Ullah et al. discovered that supplementing adult female rats with polycystic ovary syndrome (PCOS) with varying doses of GABA resulted in significant reductions in body mass index (BMI) and testosterone levels [113]. Fan et al. reported that adding GABA to growing pig diets can significantly increase the secretion levels of growth hormone (GH), melano-stimulating hormone, and thyroid stimulating hormone (TSH) [114]. GH boosts the metabolic rate of muscle cells, alters the distribution of dietary nutrients in the animal body, and enhances animal growth rate and feed conversion efficiency. In addition, GH can promote the entry of amino acids into cells through insulin-like growth factor (IGF), accelerate the synthesis of DNA and RNA, and then promote protein synthesis, and at the same time accelerate lipolysis, enhance the oxidation of fatty acids and energy supply, reduce the fat content of limb tissues, improve the lean meat rate, and improve carcass quality [115].

### 6.4. Immunoregulation

GABA can boost mucosal immunity in rats by inhibiting the secretion of somatostatin (SS) in the digestive tract [116,117]. SS possesses broad immunosuppressive effects, including a significant inhibition of immunoglobulin synthesis, particularly IgA synthesis, by up to 20% to 50%. Numerous studies indicate that GABA influences the secretion of various endocrine hormones, which have diverse immunomodulatory effects [118,119]. For instance, GH stimulates antibody and IL-2 production, activates macrophages, and enhances killer T cell generation [120]. Moreover, GABA promotes animal feeding and augments nutrient absorption and utilization, and since nutritional status significantly impacts immunity, GABA indirectly modulates the immune system through its nutritional effects.

### 6.5. Detoxification of Ammonia

Under the conditions of modern industrial and biochemical intensive feeding, livestock manure, urine, and food residues often accumulate, leading to high concentrations of harmful gases such as ammonia in the environment, posing health risks to animals and reducing productivity. GABA can react with α-ketoglutaric acid to produce glutamic acid, inhibiting the decarboxylation of glutamic acid. This effectively lowers blood ammonia concentration, facilitating the combination of more glutamic acid with ammonia to produce urea for excretion, thus mitigating ammonia toxicity and enhancing liver function [121]. Additionally, GABA can reduce NH^4+^ levels in the blood and brain, while increasing glutamine concentration. This decrease in NH^4+^ transfer from the blood to the brain suggests that GABA has a detoxifying effect on ammonia.

### 6.6. Neurotrophic Factor

In recent years, studies have revealed additional benefits of GABA, including its ability to lower blood pressure, act as an anti-convulsant, and reduce anxiety. Furthermore, GABA plays a neurotrophic role in nervous system development, potentially serving as a neurotrophic factor [122,123]. Research suggests that GABA may have therapeutic potential in treating depression through the gut–brain axis [124]. These findings position GABA as a promising neuroregenerative agent worthy of further exploration in neuropathic diseases.

### 6.7. Cardioprotection

Recent research has suggested a potential link between gut microbiota and cardiovascular diseases [125,126]. Wang et al. discovered that prophylactic supplementation of Lactobacillus Reuteri or its metabolite, GABA, can ameliorate myocardial ischemia-reperfusion injury in mice. This supplementation leads to reduced levels of myocardial injury markers, improved cardiac function, alleviated cardiac fibrosis, and suppressed inflammatory reactions. GABA primarily attenuates inflammation by decreasing the population of pro-inflammatory macrophages and inhibiting macrophage polarization toward pro-inflammatory M1 cells. Furthermore, GABA inhibits macrophage lysosomal leakage and NLRP3 inflammasome activation. This study holds significant implications for understanding the role of probiotics and their metabolites in preventing myocardial ischemia-reperfusion injury [127].

## 7. Prospects and Challenges

Future prospects for GABA production are promising and can be summarized as follows: Employing metabolic engineering and synthetic biology techniques, we can optimize the GABA synthesis pathway, enhance precursor availability, and overcome metabolic constraints to achieve higher GABA yields. By gaining a deeper understanding of the structure–function relationship of GAD enzymes from different sources, we can utilize protein engineering methods to design more efficient and specific variants. This will significantly contribute to improved GABA production. In addition to bacteria, exploring other microbial hosts such as yeast or filamentous fungi holds potential advantages in terms of safety, productivity, and substrate utilization for GABA production. Developing cost-effective and scalable fermentation processes, optimizing culture conditions, and implementing efficient downstream purification techniques are crucial for large-scale GABA production. Moreover, GABA has demonstrated potential therapeutic effects on various health conditions, including anxiety, insomnia, and hypertension. Further research on and development of GABA-based pharmaceuticals and functional foods will create new market opportunities. In conclusion, the future development of GABA production relies on continuous advancements in microbial strains, rational design of GAD enzymes, exploration of alternative hosts, process optimization, and the exploration of new applications in health and medicine. These advancements will not only meet the increasing demand for GABA but also unlock its full potential in various industries.

Microbial fermentation has emerged as a necessary approach for GABA production. However, most GABA-producing microorganisms reported so far are bacteria, which may raise safety concerns due to potential endotoxin production. Therefore, it is essential to continuously develop highly efficient and safe GABA production strains. Advancements in molecular biology, proteomics, bioinformatics, and related fields offer opportunities to obtain superior biological enzymes and secure microbial expression systems through gene editing and protein-directed modification. However, our current understanding of the structure and catalytic mechanism of glutamate decarboxylase (GAD), the enzyme responsible for GABA synthesis, is limited. This knowledge gap impedes our ability to fully comprehend GAD’s catalytic mechanism and enhance its protein structure to generate more efficient GAD variants. Therefore, it is crucial to explore GAD’s structure and analyze its catalytic mechanism from different sources to facilitate future advancements in GABA production. Furthermore, the transition from laboratory research to commercialized products is a multifaceted and lengthy endeavor, demanding attention to numerous elements such as process stability, product quality, and manufacturing expenses. Animal models have inherent limitations in mimicking human diseases, posing significant challenges for the development of GABA-targeted drugs based on these models. The GABA neurotransmitter system interacts with other neurotransmitter systems to form an intricate neural regulatory network, making it extremely difficult to decipher the mechanisms underlying GABA’s actions. In conclusion, from the development of GAD, the key enzyme for GABA synthesis, and the selection of safe and efficient strains to the industrial production of GABA and the subsequent preclinical and clinical evaluation of GABA-based drugs, every step of the process is fraught with challenges.

## Figures and Tables

**Table 1:** GAD from microorganisms.

Microorganism	Optimum Temperature (°C)	Optimum pH	References
*E. coli*	37 °C	4.8~5.0	[25]
*L. plantarum* EJ2014	30 °C	4.0~5.0	[26]
*K. marxianus* C21	35 °C	4.0	[27]
*Lb. zymae* GU240	41 °C	4.5	[28]
*E.s faecium*	45 °C	6.6	[29]
*Pyrococcus horikoshii*	60 °C	-	[30]
*E. sulfureus*	55 °C	4	[31]
*L. lactis*	55 °C	5.5	[31]
*L. senmaizukei*	40 °C	5.5	[31]
*L. brevis*	50 °C	4.5	[31]
*B. thetaiotaomicron* VPI-5482	60 °C	3.6	[32]
*L. garvieae* MJF010	35 °C	5	[33]
*M. smegmatis*	-	5.4	[34]
*Mycobacterium leprae*	37 °C	4.5	[35]

**Table 2:** GAD from plants.

Plant	Optimum Temperature (°C)	Optimum pH	References
Soybean	40 °C	5.8	[36]
Rice germ	40 °C	5.5~5.8	[37]
Potato tubers	37 °C	5.8	[38]
Apple	-	5.5–6.0	[39]
Germinated faba bean	40 °C	6.0	[40]
Wheat embryos	30 °C	5.8	[41]
Camellia sinensis	40 °C/55 °C	5.6	[42]
*Synechocystis* sp. PCC6803	30 °C	6	[43]

**Table 3:** GAD from animals.

Animal	Optimum Temperature (°C)	Optimum pH	References
Human	37 °C	6.5~7.0	[44]
*Petromyzon marinus*	27~30 °C	6.8	[45]
Mouse	37 °C	7.0	[46]
Crassostrea gigas	15–18 °C	-	[47]
crayfish	-	7–10	[48]
*Drosophila melanogaster*	-	7.5	[49]
Chick ampullary cistae	-	7.3	[50]

## Data Availability

All data generated or analyzed during this study are included in this published article.

## References

[1] Roberts E., Frankel S. (1950). γ-Aminobutyric acid in brain: Its formation from glutamic acid. J. Biol. Chem..

[2] Li H., Cao Y. (2010). Lactic Acid Bacterial Cell Factories for Gamma-Aminobutyric Acid. Amino Acids.

[3] Hao R., Schmit J.C. (1991). Purification and Characterization of Glutamate Decarboxylase from Neurospora Crassa Conidia. J. Biol. Chem..

[4] Khan M.I.R., Jalil S.U., Chopra P., Chhillar H., Ferrante A., Khan N.A., Ansari M.I. (2021). Role of GABA in Plant Growth, Development and Senescence. Plant Gene.

[5] Kinnersley A.M., Turano F.J. (2000). Gamma Aminobutyric Acid (GABA) and Plant Responses to Stress. Crit. Rev. Plant Sci..

[6] Takahashi H., Sumi M., Koshino F. (1961). Effect of γ-Aminobutyric Acid (GABA) on Normotensive or Hypertensive Rats and Men. Jpn. J. Physiol..

[7] Wassef A., Baker J., Kochan L.D. (2003). GABA and Schizophrenia: A Review of Basic Science and Clinical Studies. J. Clin. Psychopharmacol..

[8] Martins-Marques T. (2023). Cardioprotective Role of GABA-B Receptor Activation on Ventricular Arrhythmia Following Myocardial Infarction. Rev. Port. Cardiol..

[9] Hagan D.W., Ferreira S.M., Santos G.J., Phelps E.A. (2022). The Role of GABA in Islet Function. Front. Endocrinol..

[10] Martin A., Mick G.J., Choat H.M., Lunsford A.A., Tse H.M., McGwin G.G., McCormick K.L. (2022). A Randomized Trial of Oral Gamma Aminobutyric Acid (GABA) or the Combination of GABA with Glutamic Acid Decarboxylase (GAD) on Pancreatic Islet Endocrine Function in Children with Newly Diagnosed Type 1 Diabetes. Nat. Commun..

[11] Wherrett D.K., Bundy B., Becker D.J., DiMeglio L.A., Gitelman S.E., Goland R., Gottlieb P.A., Greenbaum C.J., Herold K.C., Marks J.B. (2011). Antigen-Based Therapy with Glutamic Acid Decarboxylase (GAD) Vaccine in Patients with Recent-Onset Type 1 Diabetes: A Randomised Double-Blind Trial. Lancet.

[12] Hata T., Rehman F., Hori T., Nguyen J.H. (2019). GABA, γ-Aminobutyric Acid, Protects against Severe Liver Injury. J. Surg. Res..

[13] Yang Y., Ren L., Li W., Zhang Y., Zhang S., Ge B., Yang H., Du G., Tang B., Wang H. (2023). GABAergic Signaling as a Potential Therapeutic Target in Cancers. Biomed. Pharmacother..

[14] Ngo D.-H., Vo T.S. (2019). An Updated Review on Pharmaceutical Properties of Gamma-Aminobutyric Acid. Molecules.

[15] Zhong H.-J., Wang S.-Q., Zhang R.-X., Zhuang Y.-P., Li L., Yi S.-Z., Li Y., Wu L., Ding Y., Zhang J. (2023). Supplementation with High-GABA-Producing Lactobacillus Plantarum L5 Ameliorates Essential Tremor Triggered by Decreased Gut Bacteria-Derived GABA. Transl. Neurodegener..

[16] Perucca E., White H.S., Bialer M. (2023). New GABA-Targeting Therapies for the Treatment of Seizures and Epilepsy: II. Treatments in Clinical Development. CNS Drugs.

[17] Felice D., Cryan J.F., O’Leary O.F. (2022). GABAB Receptors: Anxiety and Mood Disorders. Curr. Top. Behav. Neurosci..

[18] Schousboe A., Madsen K.K., Barker-Haliski M.L., White H.S. (2014). The GABA Synapse as a Target for Antiepileptic Drugs: A Historical Overview Focused on GABA Transporters. Neurochem. Res..

[19] Erlander M.G., Tobin A.J. (1991). The Structural and Functional Heterogeneity of Glutamic Acid Decarboxylase: A Review. Neurochem. Res..

[20] Zhang Q.-F., Hu S., Zhao W.-R., Huang J., Mei J.-Q., Mei L.-H. (2020). Parallel Strategy Increases the Thermostability and Activity of Glutamate Decarboxylase. Molecules.

[21] Patel A.K., Singhania R.R., Pandey A., Brahmachari G. (2017). Chapter 2—Production, Purification, and Application of Microbial Enzymes. Biotechnology of Microbial Enzymes.

[22] Xiong W., Liu B., Shen Y., Jing K., Savage T.R. (2021). Protein Engineering Design from Directed Evolution to de Novo Synthesis. Biochem. Eng. J..

[23] Wahab M.K.H.A., El-Enshasy H.A., Bakar F.D.A., Murad A.M.A., Illias R.M. (2019). Improvement of Cross-Linking and Stability on Cross-Linked Enzyme Aggregate (CLEA)-Xylanase by Protein Surface Engineering. Process Biochem..

[24] Ueno H. (2000). Enzymatic and Structural Aspects on Glutamate Decarboxylase. J. Mol. Catal. B Enzym..

[25] Najjar V.A., Fisher J. (1954). Studies on l-glutamic acid decarboxylase from escherichia coli. J. Biol. Chem..

[26] Park S.J., Kim D.H., Kang H.J., Shin M., Yang S.-Y., Yang J., Jung Y.H. (2021). Enhanced Production of γ-Aminobutyric Acid (GABA) Using Lactobacillus Plantarum EJ2014 with Simple Medium Composition. LWT.

[27] Zhang L., Yue Y., Wang X., Dai W., Piao C., Yu H. (2022). Optimization of Fermentation for γ-Aminobutyric Acid (GABA) Production by Yeast Kluyveromyces Marxianus C21 in Okara (Soybean Residue). Bioprocess. Biosyst. Eng..

[28] Park J.Y., Jeong S.-J., Kim J.H. (2014). Characterization of a Glutamate Decarboxylase (GAD) Gene from Lactobacillus Zymae. Biotechnol. Lett..

[29] Yarabbi H., Mortazavi S.A., Yavarmanesh M., Javadmanesh A. (2021). Molecular Cloning, Gene Overexpression and Characterization of Glutamate Decarboxylase from *Enterococcus Faecium* DO. LWT.

[30] Somasundaram S., Jeong J., Kumaravel A., Hong S.H. (2021). Whole-Cell Display of Pyrococcus Horikoshii Glutamate Decarboxylase in Escherichia Coli for High-Titer Extracellular Gamma-Aminobutyric Acid Production. J. Ind. Microbiol. Biotechnol..

[31] Tang C.-D., Li X., Shi H.-L., Jia Y.-Y., Dong Z.-X., Jiao Z.-J., Wang L.-F., Xu J.-H., Yao L.-G., Kan Y.-C. (2020). Efficient Expression of Novel Glutamate Decarboxylases and High Level Production of γ-Aminobutyric Acid Catalyzed by Engineered Escherichia Coli. Int. J. Biol. Macromol..

[32] Liu S., Wen B., Du G., Wang Y., Ma X., Yu H., Zhang J., Fan S., Zhou H., Xin F. (2023). Coordinated Regulation of Bacteroides Thetaiotaomicron Glutamate Decarboxylase Activity by Multiple Elements under Different pH. Food Chem..

[33] Lim H.J., Jung D.-H., Cho E.-S., Seo M.-J. (2022). Expression, Purification, and Characterization of Glutamate Decarboxylase from Human Gut-Originated Lactococcus Garvieae MJF010. World J. Microbiol. Biotechnol..

[34] Li Y., Chen G., Ge F., Dang T., Ren Y., Zeng B., Li W. (2022). Characterization and Mutagenesis of a Novel Mycobacterium Smegmatis-Derived Glutamate Decarboxylase Active at Neutral pH. World J. Microbiol. Biotechnol..

[35] Prabhakaran K., Harris E.B., Kirchheimer W.F. (1983). Glutamic Acid Decarboxylase in Mycobacterium Leprae. Arch. Microbiol..

[36] Snedden W.A., Arazi T., Fromm H., Shelp B.J. (1995). Calcium/Calmodulin Activation of Soybean Glutamate Decarboxylase. Plant Physiol..

[37] Zhang H., Yao H., Chen F., Wang X. (2007). Purification and Characterization of Glutamate Decarboxylase from Rice Germ. Food Chem..

[38] Satyanarayan V., Nair P.M. (1985). Purification and Characterization of Glutamate Decarboxylase from Solanum Tuberosum. Eur. J. Biochem..

[39] Trobacher C.P., Zarei A., Liu J., Clark S.M., Bozzo G.G., Shelp B.J. (2013). Calmodulin-Dependent and Calmodulin-Independent Glutamate Decarboxylases in Apple Fruit. BMC Plant Biol..

[40] Yang R., Yin Y., Guo Q., Gu Z. (2013). Purification, Properties and cDNA Cloning of Glutamate Decarboxylase in Germinated Faba Bean (*Vicia faba* L.). Food Chem..

[41] On the Nature of Glutamic Acid Decarboxylase in Wheat Embryos 12|Plant Physiology|Oxford Academic. https://academic.oup.com/plphys/article/35/1/68/6089595?login=false.

[42] Mei X., Xu X., Yang Z. (2020). Characterization of Two Tea Glutamate Decarboxylase Isoforms Involved in GABA Production. Food Chem..

[43] Kanwal S., Incharoensakdi A. (2016). Characterization of Glutamate Decarboxylase from *Synechocystis* Sp. PCC6803 and Its Role in Nitrogen Metabolism. Plant Physiol. Biochem..

[44] Hamel E., Krause D.N., Roberts E. (1982). Characterization of Glutamic Acid Decarboxylase Activity in Cerebral Blood Vessels. J. Neurochem..

[45] Wald U., Selzer M.E., Krieger N.R. (1981). Glutamic Acid Decarboxylase in Sea Lamprey (*Petromyzon marinus*): Characterization, Localization, and Developmental Changes. J. Neurochem..

[46] Wu J.-Y., Matsuda T., Roberts E. (1973). Purification and Characterization of Glutamate Decarboxylase from Mouse Brain. J. Biol. Chem..

[47] Li M., Wang L., Qiu L., Wang W., Xin L., Xu J., Wang H., Song L. (2016). A Glutamic Acid Decarboxylase (CgGAD) Highly Expressed in Hemocytes of Pacific Oyster Crassostrea Gigas. Dev. Comp. Immunol..

[48] Grossfeld R.M., Yancey S.W., Baxter C.F. (1984). Assay and Properties of Glutamic Acid Decarboxylase in Homogenates of Crayfish Nervous Tissue. Comp. Biochem. Physiol. Part B Comp. Biochem..

[49] Chude O., Roberts E., Wu J. (1979). Partial purification of *drosophila* glutamate decarboxylase. J. Neurochem..

[50] Meza G. (1984). Some Characteristics of Glutamic Acid Decarboxylase of Chick Ampullary Cristae. J. Neurochem..

[51] Steffen-Munsberg F., Vickers C., Kohls H., Land H., Mallin H., Nobili A., Skalden L., van den Bergh T., Joosten H.-J., Berglund P. (2015). Bioinformatic Analysis of a PLP-Dependent Enzyme Superfamily Suitable for Biocatalytic Applications. Biotechnol. Adv..

[52] Saxena V.K., Vedamurthy G.V., Singh R. (2022). A Novel Concept of Pyridoxal 5’-Phosphate Permeability in E.Coli for Modulating the Heterologous Expression of PLP Dependent Proteins. Process Biochem..

[53] Du Y.-L., Ryan K.S. (2019). Pyridoxal Phosphate-Dependent Reactions in the Biosynthesis of Natural Products. Nat. Prod. Rep..

[54] Wang C., Zhu R., Sun H., Li B. (2013). Quantum Chemistry Studies of the Catalysis Mechanism Differences between the Two Isoforms of Glutamic Acid Decarboxylase. J. Mol. Model..

[55] Biase D.D., Tramonti A., John R.A., Bossa F. (1996). Isolation, Overexpression, and Biochemical Characterization of the Two Isoforms of Glutamic Acid Decarboxylase fromEscherichia Coli. Protein Expr. Purif..

[56] De Biase D., Tramonti A., Bossa F., Visca P. (1999). The Response to Stationary-Phase Stress Conditions in *Escherichia coli*: Role and Regulation of the Glutamic Acid Decarboxylase System. Mol. Microbiol..

[57] Capitani G., Biase D.D., Aurizi C., Gut H., Bossa F., Grütter M.G. (2003). Crystal Structure and Functional Analysis of Escherichia Coli Glutamate Decarboxylase. EMBO J..

[58] Smith A.M., Brown W.C., Harms E., Smith J.L. (2015). Crystal Structures Capture Three States in the Catalytic Cycle of a Pyridoxal Phosphate (PLP) Synthase. J. Biol. Chem..

[59] Huang J., Fang H., Gai Z.-C., Mei J.-Q., Li J.-N., Hu S., Lv C.-J., Zhao W.-R., Mei L.-H. (2018). Lactobacillus Brevis CGMCC 1306 Glutamate Decarboxylase: Crystal Structure and Functional Analysis. Biochem. Biophys. Res. Commun..

[60] Grzechowiak M., Sliwiak J., Jaskolski M., Ruszkowski M. (2023). Structural and Functional Studies of Arabidopsis Thaliana Glutamate Dehydrogenase Isoform 2 Demonstrate Enzyme Dynamics and Identify Its Calcium Binding Site. Plant Physiol. Biochem..

[61] Arazi T., Baum G., Snedden W.A., Shelp B.J., Fromm H. (1995). Molecular and Biochemical Analysis of Calmodulin Interactions with the Calmodulin-Binding Domain of Plant Glutamate Decarboxylase. Plant Physiol..

[62] Gut H., Dominici P., Pilati S., Astegno A., Petoukhov M.V., Svergun D.I., Grütter M.G., Capitani G. (2009). A Common Structural Basis for pH- and Calmodulin-Mediated Regulation in Plant Glutamate Decarboxylase. J. Mol. Biol..

[63] Astegno A., Capitani G., Dominici P. (2015). Functional Roles of the Hexamer Organization of Plant Glutamate Decarboxylase. Biochim. Biophys. Acta (BBA) Proteins Proteom..

[64] Zik M., Arazi T., Snedden W.A., Fromm H. (1998). Two Isoforms of Glutamate Decarboxylase in Arabidopsis Are Regulated by Calcium/Calmodulin and Differ in Organ Distribution. Plant Mol. Biol..

[65] Tokumitsu H., Sakagami H. (2022). Molecular Mechanisms Underlying Ca^2+^/Calmodulin-Dependent Protein Kinase Kinase Signal Transduction. Int. J. Mol. Sci..

[66] Teresinski H.J., Hau B., Symonds K., Kilburn R., Munro K.A., Doner N.M., Mullen R., Li V.H., Snedden W.A. (2023). Arabidopsis Calmodulin-like Proteins CML13 and CML14 Interact with Proteins That Have IQ Domains. Plant Cell Environ..

[67] Ishida H., Rainaldi M., Vogel H.J. (2009). Structural Studies of Soybean Calmodulin Isoform 4 Bound to the Calmodulin-Binding Domain of Tobacco Mitogen-Activated Protein Kinase Phosphatase-1 Provide Insights into a Sequential Target Binding Mode*. J. Biol. Chem..

[68] Pigott T., McPeak A., de Chastelain A., DeMayo M.M., Rasic N., Rayner L., Noel M., Miller J.V., Harris A.D. (2023). Changes in Brain GABA and Glutamate and Improvements in Physical Functioning Following Intensive Pain Rehabilitation in Youth with Chronic Pain. J. Pain..

[69] Almeida M.R., Mabasa L., Crane C., Park C.S., Venâncio V.P., Bianchi M.L.P., Antunes L.M.G. (2016). Maternal Vitamin B _6_ Deficient or Supplemented Diets on Expression of Genes Related to GABAergic, Serotonergic, or Glutamatergic Pathways in Hippocampus of Rat Dams and Their Offspring. Mol. Nutr. Food Res..

[70] Kakizaki T., Oriuchi N., Yanagawa Y. (2015). GAD65/GAD67 Double Knockout Mice Exhibit Intermediate Severity in Both Cleft Palate and Omphalocele Compared with GAD67 Knockout and VGAT Knockout Mice. Neuroscience.

[71] Reetz A., Solimena M., Matteoli M., Folli F., Takei K., De Camilli P. (1991). GABA and Pancreatic Beta-Cells: Colocalization of Glutamic Acid Decarboxylase (GAD) and GABA with Synaptic-like Microvesicles Suggests Their Role in GABA Storage and Secretion. EMBO J..

[72] Szpręgiel I., Wrońska D., Kmiecik M., Pałka S., Kania B.F. (2021). Glutamic Acid Decarboxylase Concentration Changes in Response to Stress and Altered Availability of Glutamic Acid in Rabbit (*Oryctolagus cuniculus*) Brain Limbic Structures. Animals.

[73] Fenalti G., Law R.H.P., Buckle A.M., Langendorf C., Tuck K., Rosado C.J., Faux N.G., Mahmood K., Hampe C.S., Banga J.P. (2007). GABA Production by Glutamic Acid Decarboxylase Is Regulated by a Dynamic Catalytic Loop. Nat. Struct. Mol. Biol..

[74] Langendorf C.G., Tuck K.L., Key T.L.G., Fenalti G., Pike R.N., Rosado C.J., Wong A.S.M., Buckle A.M., Law R.H.P., Whisstock J.C. (2013). Structural Characterization of the Mechanism through Which Human Glutamic Acid Decarboxylase Auto-Activates. Biosci. Rep..

[75] Zeng C., Lei D., Lu Y., Huang Q., Wu Y., Yang S., Wu Y. (2023). Parvalbumin in the Metabolic Pathway of Glutamate and γ-Aminobutyric Acid: Influence on Expression of GAD65 and GAD67. Arch. Biochem. Biophys..

[76] Lazarus M.S., Krishnan K., Huang Z.J. (2015). GAD67 Deficiency in Parvalbumin Interneurons Produces Deficits in Inhibitory Transmission and Network Disinhibition in Mouse Prefrontal Cortex. Cereb. Cortex.

[77] Gu X., Zhang R., Zhao J., Li C., Guo T., Yang S., Han T., Kong J. (2022). Fast-Acidification Promotes GABA Synthesis in Response to Acid Stress in Streptococcus Thermophilus. LWT.

[78] Sarasa S.B., Mahendran R., Muthusamy G., Thankappan B., Selta D.R.F., Angayarkanni J. (2020). A Brief Review on the Non-Protein Amino Acid, Gamma-Amino Butyric Acid (GABA): Its Production and Role in Microbes. Curr. Microbiol..

[79] Yin Y., Yang R., Guo Q., Gu Z. (2014). NaCl Stress and Supplemental CaCl2 Regulating GABA Metabolism Pathways in Germinating Soybean. Eur. Food Res. Technol..

[80] Sun Y., Mehmood A., Battino M., Xiao J., Chen X. (2022). Enrichment of Gamma-Aminobutyric Acid in Foods: From Conventional Methods to Innovative Technologies. Food Res. Int..

[81] Li E., Luo X., Liao S., Shen W., Li Q., Liu F., Zou Y. (2018). Accumulation of Γ-aminobutyric Acid during Cold Storage in Mulberry Leaves. Int. J. Food Sci. Tech..

[82] Pannerchelvan S., Rios-Solis L., Wong F.W.F., Zaidan U.H., Wasoh H., Mohamed M.S., Tan J.S., Mohamad R., Halim M. (2023). Strategies for Improvement of Gamma-Aminobutyric Acid (GABA) Biosynthesis via Lactic Acid Bacteria (LAB) Fermentation. Food Funct..

[83] Soma Y., Fujiwara Y., Nakagawa T., Tsuruno K., Hanai T. (2017). Reconstruction of a Metabolic Regulatory Network in Escherichia Coli for Purposeful Switching from Cell Growth Mode to Production Mode in Direct GABA Fermentation from Glucose. Metab. Eng..

[84] Wei L., Zhao J., Wang Y., Gao J., Du M., Zhang Y., Xu N., Du H., Ju J., Liu Q. (2022). Engineering of *Corynebacterium Glutamicum* for High-Level γ-Aminobutyric Acid Production from Glycerol by Dynamic Metabolic Control. Metab. Eng..

[85] Lyu J.-F., Lyu C.-J., Cao J.-R., Mei J.-Q., Hu S., Zhao W.-R., Xu T.-Y., Wang Y.-T., Wang D.-L., Huang J. (2022). High Level Production of γ-Aminobutyric Acid in Engineered Escherichia Coli by Refactoring the Glutamate Decarboxylase. Process Biochem..

[86] Yang L., Zhang X., Chen J., Zhang Y., Feng Z. (2023). Expanding the pH Range of Glutamate Decarboxylase from L. Pltarum LC84 by Site-Directed Mutagenesis. Front. Bioeng. Biotechnol..

[87] Fan L.-Q., Li M.-W., Qiu Y., Chen Q., Jiang S.-J., Shang Y.-J., Zhao L.-M. (2018). Increasing Thermal Stability of Glutamate Decarboxylase from Escherichia. Coli by Site-Directed Saturation Mutagenesis and Its Application in GABA Production. J. Biotechnol..

[88] Li H., Li B., Gao L., Ge R., Cui X., Zhou J., Li Z. (2023). Gamma-Aminobutyric Acid (GABA) Promotes Characteristics of Levilactobacillus Sp. LB-2. LWT.

[89] Park S., Sohn Y.J., Park S.J., Choi J. (2020). Effect of DR1558, a Deinococcus Radiodurans Response Regulator, on the Production of GABA in the Recombinant Escherichia Coli under Low pH Conditions. Microb. Cell Fact..

[90] Edalatian Dovom M.R., Habibi Najafi M.B., Rahnama Vosough P., Norouzi N., Ebadi Nezhad S.J., Mayo B. (2023). Screening of Lactic Acid Bacteria Strains Isolated from Iranian Traditional Dairy Products for GABA Production and Optimization by Response Surface Methodology. Sci. Rep..

[91] Asun A.C., Lin S.-T., Ng H.S., Lan J.C.-W. (2022). Production of Gamma-Aminobutyric Acid (GABA) by Bacillus Subtilis BBEL02 Fermentation Using Nitrogen-Rich Industrial Wastes as Crude Feedstocks. Biochem. Eng. J..

[92] Villegas J.M., Brown L., Savoy de Giori G., Hebert E.M. (2016). Optimization of Batch Culture Conditions for GABA Production by Lactobacillus Brevis CRL 1942, Isolated from Quinoa Sourdough. LWT Food Sci. Technol..

[93] Laroute V., Mazzoli R., Loubière P., Pessione E., Cocaign-Bousquet M. (2021). Environmental Conditions Affecting GABA Production in Lactococcus Lactis NCDO 2118. Microorganisms.

[94] Xue C., Ng I.-S. (2023). Investigation of enzymatic quality and quantity using pyridoxal 5′-phosphate (PLP) regeneration system as a decoy in escherichia coli. Int. J. Biol. Macromol..

[95] Yang X., Huo X., Tang Y., Zhao M., Tao Y., Huang J., Ke C. (2023). Integrating Enzyme Evolution and Metabolic Engineering to Improve the Productivity of Γ-Aminobutyric Acid by Whole-Cell Biosynthesis in Escherichia Coli. J. Agric. Food. Chem..

[96] Zhang L., Zhao H., Gan M., Jin Y., Gao X., Chen Q., Guan J., Wang Z. (2011). Application of Simultaneous Saccharification and Fermentation (SSF) from Viscosity Reducing of Raw Sweet Potato for Bioethanol Production at Laboratory, Pilot and Industrial Scales. Bioresour. Technol..

[97] Han J., Zhao X., Zhao X., Wang Q., Li P., Gu Q. (2023). Microbial-Derived γ-Aminobutyric Acid: Synthesis, Purification, Physiological Function, and Applications. J. Agric. Food. Chem..

[98] Vosoughi A., Zendehdel M., Zarei H., Hassanpour S. (2024). Central Effects of the Serotoninergic, GABAergic, and Cholecystokinin Systems on Neuropeptide VF (NPVF)-Induced Hypophagia and Feeding Behavior in Neonatal Broiler Chicken. Neurosci. Lett..

[99] Pu S., Jain M.R., Horvath T.L., Diano S., Kalra P.S., Kalra S.P. (1999). Interactions between Neuropeptide Y and γ -Aminobutyric Acid in Stimulation of Feeding: A Morphological and Pharmacological Analysis*. Endocrinology.

[100] Wu X., Saiyidi N.M., Nie B., Han B., Yang K. (2015). Effects of γ-Aminobutyric Acid on the Growth Performance and Blood Indicators in Sheep with 6-8 Months Old. China Feed.

[101] Chen Q., Liang J. (2012). The Effect of γ-aminobutyric on Growth Performance and Serum Levels of Protein and Enzyme Activity in Weaned Piglets. Chin. Agric. Sci. Bull..

[102] Fan Z.Y., Deng J.P., Liu G.H., Cai H.Y., He J.H., Wu M.X., Zhen F.Y. (2007). Effects of γ-Aminobutyric Acid on the Performance and Internal Hormone Levels in Growing Pigs. Chin. J. Anim. Nutr..

[103] Tanaka C. (1985). γ-aminobutyric acid in peripheral tissues. Life Sci..

[104] Xiang-Gui X., Zai-Fu Y., Shen-He H.A., Xiao-Hong Y. (2001). Promotive Effects of Gaba on Acid Secretion from Isolated Mouse Stomach In Vitro. Acta Zool. Sin..

[105] Nakajima K., Tooyama I., Kuriyama K., Kimura H. (1996). Immunohistochemical Demonstration of GABAB Receptors in the Rat Gastrointestinal Tract. Neurochem. Res..

[106] Auteri M., Zizzo M., Serio R. (2015). The GABAergic System and the Gastrointestinal Physiopathology. Curr. Pharm. Des..

[107] Zhang A., Jiang X., Ge Y., Xu Q., Li Z., Tang H., Cao D., Zhang D. (2022). The Effects of GABA-Rich Adzuki Beans on Glycolipid Metabolism, as Well as Intestinal Flora, in Type 2 Diabetic Mice. Front. Nutr..

[108] Zhao Y., Wang J., Wang H., Huang Y., Qi M., Liao S., Bin P., Yin Y. (2020). Effects of GABA Supplementation on Intestinal SIgA Secretion and Gut Microbiota in the Healthy and ETEC-Infected Weanling Piglets. Mediat. Inflamm..

[109] Roth F.C., Draguhn A. (2012). GABA Metabolism and Transport: Effects on Synaptic Efficacy. Neural Plast..

[110] Abdel-Moneim A.-M.E., Shehata A.M., Khidr R.E., Paswan V.K., Ibrahim N.S., El-Ghoul A.A., Aldhumri S.A., Gabr S.A., Mesalam N.M., Elbaz A.M. (2021). Nutritional Manipulation to Combat Heat Stress in Poultry—A Comprehensive Review. J. Therm. Biol..

[111] Al Wakeel R.A., Shukry M., Abdel Azeez A., Mahmoud S., Saad M.F. (2017). Alleviation by Gamma Amino Butyric Acid Supplementation of Chronic Heat Stress-Induced Degenerative Changes in Jejunum in Commercial Broiler Chickens. Ann. Ny. Acad. Sci..

[112] Park K.-T., Oh M., Joo Y., Han J.-K. (2023). Effects of Gamma Aminobutyric Acid on Performance, Blood Cell of Broiler Subjected to Multi-Stress Environments. Anim. Biosci..

[113] Ullah A., Jahan S., Razak S., Pirzada M., Ullah H., Almajwal A., Rauf N., Afsar T. (2017). Protective Effects of GABA against Metabolic and Reproductive Disturbances in Letrozole Induced Polycystic Ovarian Syndrome in Rats. J. Ovarian Res..

[114] Bi C., Yin J., Yang W., Shi B., Shan A. (2020). Effects of Dietary γ-Aminobutyric Acid Supplementation on Antioxidant Status, Blood Hormones and Meat Quality in Growing-Finishing Pigs Undergoing Transport Stress. J. Anim. Physiol. Anim. Nutr..

[115] Zhou X., Xiao R., Long D., Xie Y., Jiang S. (2016). Effects of γ-aminobutyric Acid on Growth Performance and Meat Quality of Finishing Pigs under the Hot and Hu-mid Conditions. Chin. J. Anim. Sci..

[116] Koop H., Arnold R. (1986). Control of Rat Gastric Somatostatin Release by γ-Aminobutyric Acid (GABA). Horm. Metab. Res..

[117] Harty R.F., Franklin P.A. (1983). GABA Affects the Release of Gastrin and Somatostatin from Rat Antral Mucosa. Nature.

[118] Ferone D., Van Hagen P.M., Semino C., Dalm V.A., Barreca A., Colao A., Lamberts S.W.J., Minuto F., Hofland L.J. (2004). Somatostatin Receptor Distribution and Function in Immune System. Digest. Liver Dis..

[119] Wiens S.C., Trudeau V.L. (2006). Thyroid Hormone and γ-Aminobutyric Acid (GABA) Interactions in Neuroendocrine Systems. Comp. Biochem. Physiol. A Mol. Integr. Physiol..

[120] Huang Z., Xiao L., Xiao Y., Chen C. (2022). The Modulatory Role of Growth Hormone in Inflammation and Macrophage Activation. Endocrinology.

[121] Paul V. (2003). Inhibition of Acute Hyperammonemia-Induced Convulsions by Systemically Administered Gamma Aminobutyric Acid in Rats. Pharmacol. Biochem. Behav..

[122] Fiszman M.L., Schousboe A. (2004). Role of Calcium and Kinases on the Neurotrophic Effect Induced by Γ-aminobutyric Acid. J. Neurosci. Res..

[123] Mumtaz F., Khan M.I., Zubair M., Dehpour A.R. (2018). Neurobiology and Consequences of Social Isolation Stress in Animal Model—A Comprehensive Review. Biomed. Pharmacother..

[124] Liwinski T., Lang U.E., Brühl A.B., Schneider E. (2023). Exploring the Therapeutic Potential of Gamma-Aminobutyric Acid in Stress and Depressive Disorders through the Gut–Brain Axis. Biomedicines.

[125] Witkowski M., Weeks T.L., Hazen S.L. (2020). Gut Microbiota and Cardiovascular Disease. Circ. Res..

[126] Tang W.H.W., Kitai T., Hazen S.L. (2017). Gut Microbiota in Cardiovascular Health and Disease. Circ. Res..

[127] Wang J., Zhang H., Yuan H., Chen S., Yu Y., Zhang X., Gao Z., Du H., Li W., Wang Y. (2024). Prophylactic Supplementation with *Lactobacillus Reuteri* or Its Metabolite GABA Protects against Acute Ischemic Cardiac Injury. Adv. Sci..

